# An Improved Battery

**Published:** 1869-09

**Authors:** 


An Improved Battery.—We have recorded so many improvements (as they are all called) in galvanic batteries, that the number and variety become bewildering. The last we meet with is that suggested by Bottger, who proposes to substitute metallic antimony for carbon. An amalgamated zinc plate is immersed in a strong solution common salt and sulphate of magnesia. The antimony, like the carbon, is placed in a porous pot, but the liquid used is dilute sulphuric acid. A combination of this arrangement is said to give a stronger and more lasting current than a cell of Daniel's battery.—*Mechanics' Magazine.*

